# A Skin Lipidomics Study Reveals the Therapeutic Effects of Tanshinones in a Rat Model of Acne

**DOI:** 10.3389/fphar.2021.675659

**Published:** 2021-06-10

**Authors:** Tingting Chen, Zhaoming Zhu, Qunqun Du, Zhuxian Wang, Wenfeng Wu, Yaqi Xue, Yuan Wang, Yufan Wu, Quanfu Zeng, Cuiping Jiang, Chunyan Shen, Li Liu, Hongxia Zhu, Qiang Liu

**Affiliations:** ^1^ School of Traditional Chinese Medicine, Southern Medical University, Guangzhou, China; ^2^ Integrated Hospital of Traditional Chinese Medicine, Southern Medical University, Guangzhou, China

**Keywords:** tanshinone, skin lipidomics, acne, glycerophospholipid metabolism, sphingolipid metabolism

## Abstract

Tanshinone (TAN), a class of bioactive components in traditional Chinese medicinal plant Salvia miltiorrhiza, has antibacterial and anti-inflammatory effects, can enhance blood circulation, remove blood stasis, and promote wound healing. For these reasons it has been developed as a drug to treat acne. The purpose of this study was to evaluate the therapeutic effects of TAN in rats with oleic acid-induced acne and to explore its possible mechanisms of action through the identification of potential lipid biomarkers. In this study, a rat model of acne was established by applying 0.5 ml of 80% oleic acid to rats’ back skin. The potential metabolites and targets involved in the anti-acne effects of TAN were predicted using lipidomics. The results indicate that TAN has therapeutic efficacy for acne, as supported by the results of the histological analyses and biochemical index assays for interleukin (IL)-8, IL-6, IL-β and tumor necrosis factor alpha. The orthogonal projection of latent structure discriminant analysis score was used to analyze the lipidomic profiles between control and acne rats. Ninety-six potential biomarkers were identified in the skin samples of the acne rats. These biomarkers were mainly related to glycerophospholipid and sphingolipid metabolism, and the regulation of their dysfunction is thought to be a possible therapeutic mechanism of action of TAN on acne.

## Introduction

*Salvia miltiorrhiza* Bunge, a traditional Chinese medicine plant, was first recorded by Zhu Di in “Puji Fang” for acne treatment. Tanshinones (TANs), a class of chemical compounds and the major active component in *Salvia miltiorrhiza* Bunge, has also been reported as a treatment for cardiovascular disease (Weng et al., 2013; Maione et al., 2014; Maione et al., 2015; Jia et al., 2016). TAN has been recently shown to improve the condition of the facial skin by helping to reduce scarring through improving blood circulation and promoting cell metabolism (Li et al., 2016). Pharmacological studies show that cryptotanshinone has anti-acne effects (Yu et al., 2016) and Tanshinone IIA has an inhibitory effect on the growth of *Propionibacterium acnes* (Li and Zhou, 2018). In addition, TANs have exhibited anti-oxidant (Fang et al., 2008; Li et al., 2008; Li et al., 2013), anti-bacterial, anti-inflammatory (Bai et al., 2008; Tang et al., 2011; Chen et al., 2019), and anti-fibrosis (Jiang et al., 2019) effects. Furthermore, a recent report has found TAN to regulate tissue repair (Bernut et al., 2020). Consequently, TAN has been included in the research and development of cosmetic materials (Tao et al., 2013; Natshch et al., 2019).

Acne is one of the most common skin diseases and affects approximately 85% of the population at some point in their lifetimes (Thiboutot et al., 2018). Its pathogenesis is multifactorial and includes an increased level and sensitivity of androgen receptors (Barros and Thiboutot, 2017), excessive sebum secretion (Li et al., 2017), abnormal ductal keratosis of sebaceous glands in hair follicles (Das and Reynolds, 2014), and the colonization of *Propionibacterium acnes*, leading to an inflammatory response in the skin (Zouboulis, 2009). A pivotal factor in the aetiopathogenesis of acne is the quantitative and qualitative modification of skin lipids. Skin lipids play a significant role in the occurrence of acne lesions (Makrantonaki et al., 2011), as they can dramatically influence skin condition via different mechanisms, such as maintaining physical chemistry function, biochemistry function, and microecology function (Jia et al., 2018).

Lipidomics, a branch of metabonomics, is the study of the properties of all lipid molecules in living organisms (Han and Gross, 2003). It enables the analysis of lipids by quantifying changes in individual lipids, subgroups and molecular species. Liquid chromatography-mass spectrometry (LC-MS) is an analysis technique that allows for the comprehensive analysis of lipids and molecular types, including the lipids in lipid cells and keratinocytes (Kofeler et al., 2012; Zhao et al., 2014). In this study, changes in the lipidomic profile of the skin were analyzed using the ultra-performance liquid chromatography (UPLC)-Orbitrap MS system. Similar studies have been performed with respect to cancer, metabolic syndrome and skin diseases (Camera et al., 2016). UPLC-quadruple time-of-flight-MS is a high-resolution MS technique that can analyze the complete structure of a lipid species (van Smeden et al., 2014).

## Material and Methods

### Chemicals and Reagents

MS-grade methanol, MS-grade acetonitrile and high-performance liquid chromatography (HPLC)-grade 2-propanol were purchased from Thermo Fisher. HPLC-grade formic acid and HPLC-grade ammonium formate were purchased from Sigma. HPLC-grade acetonitrile was purchased from Merck KGaA (Darmstadt, Germany). Analytical-grade pure phosphoric acid was purchased from Guangzhou Chemical Reagent Factory. Reference standards of Tanshinone I, Crytotanshinone, Tanshinone IIA (purity >98%) were purchased from the National Institutes for Food and Drug Control (Beijing, China). Diydrotanshinone I (purity >98%) was purchased from Guangzhou Qiyun Biotechnology Co., Ltd. (Guangdong, China).

### TAN and TAN Gel Preparation

TANs are major active components of the dried root of *Salvia miltiorrhiza* Bunge. The *Salvia miltiorrhiza* Bunge pieces were purchased from Guangzhou Weida Chinese Medicine Decoction Pieces Co. Ltd., (Guangdong, China), Batch number: 201909-3. TANs were extracted and isolated from the pieces through solvent extraction. First, the pieces were pulverized into smaller, coarse granules, weighed and refluxed in 95% ethanol (1:10 w/v) for three times, 2 h each time, and filtered (Shen et al., 2017b). The filtrates were combined, with ethanol removed by decompression, resulting in a thick, concentrated filtrate paste with relative density of 1.30–1.35 (60°C). This paste was washed in hot water until colorless, dried at 80°C, and crushed into a fine powder.

The TAN gel was produced by dissolving this powder in ethanol, adding carbomer, water and triethanolamine. The TAN gel (100 mg TAN in 1 g gel) was red-brown in color and transparent.

### Qualitative and Quantitative Evaluation of TAN

The qualitative and quantitative analysis of TAN were used UHPLC-Orbitrap-MS instrument and HPLC with an UV detection system, respectively. The specific UHPLC-ESI-MS and HPLC methods used are included in the Supplementary Material.

### Experimental Animals and Acne Model

All animal were approved by the Animal Care and Use committee of the Southern Medical University (Approval No. L2019036, Guangdong, China). Adult male Sprague Dawley rats (220 ± 20 g) were purchased from the Experimental Animals Center of Southern Medical University. The rats were housed at a humidity of 40–65%, a temperature of 19–23 °C and a 12 h light-dark cycle (Jiang et al., 2007).

The rats were allocated into experimental groups through a randomized block design. All experimental procedures and data analyses were conducted in a blinded fashion. After one week of acclimatization, the rats were randomly selected as control group (C, *n* = 8) or model group. The acne rat model was established by applying oleic acid. Briefly, rats were anesthetized using intraperitoneal injections of 3% sodium pentobarbital (Sigma-Aldrich, United States). Their back hair was depilated about 4 cm^2^. 0.5 ml of 80% oleic acid (Aladdin Bio-Chem Technology, Shanghai, China) was applied to the back skin once daily for 14 days. Once the acne model was established, the acne rats were divided into the acne model group (M, *n* = 8) and the TAN gel administration group (T, *n* = 8) according to a random number control table. The rats in the TAN gel group received a dose of 1.0 g d^−1^ for 7 days. The rats in the Control and Model groups received no TAN gel treatment.

### Histopathological Examination

Sections of skin tissues from the rats were fixed in 4% formalin, embedded in paraffin, and sliced into 3–5 μm thick sections. The sections were stained with hematoxylin-eosin for histopathological analysis and observed under a light microscope (BX53; Olympus).

### Examination of Serum Inflammatory Factors

Serum samples were centrifuged and the supernatants assayed for cytokine release using the Cytokine RAT interleukin (IL)-8, IL-6, IL-1β, and tumor necrosis factor alpha (TNF-α) Kit. The detection was conducted according to the kit manual (Shen et al., 2017a).

### Lipid Extraction and Analysis

Lipids were extracted using the methyl tert-butyl ether (MTBE) method. Briefly, the skin samples were homogenized in 200 µl water and 240 µl methanol. MTBE (800 µl) was added to the homogenized sample, which was then ultrasonicated at 4°C for 20 min and afterward left to stand at room temperature for 30 min. The solution was centrifuged at 14,000 g at 10°C for 15 min, after which the upper layer was collected and dried with nitrogen. The lipid extracts were re-dissolved for analysis using 200 µl of a 90% isopropanol/acetonitrile mixture.

Q-Exactive Plus mass spectrometer (Thermo Fisher Scientific) was connected to an UHPLC Nexera LC-30A (SHIMADZU) via an electrospray ionization ion (ESI) source for the lipid analysis. The chromatographic conditions were shown in Supplementary Material.

### Data Analysis and Presentation

“LipidSearch” was used to carry out peak recognition, peak extraction, and lipid identification (secondary identification) of the lipid molecules and internal standard lipid molecules. Both the precursor tolerance and product tolerance were set at 5 ppm, and the product ion threshold was set at 5%.

Simca 14.1 software (Umetrics AB, Umea, Sweden) was used for the multivariate analysis, including the principal component analysis (PCA) and orthogonal partial least-squares discrimination analysis (OPLS-DA) (Park et al., 2020). All lipid species and subclasses were found to have equal variances and were analyzed using variation multiple analysis and t-tests.

### Statistical Analysis

The experimental data were expressed as mean ± standard deviation (S.D), unless otherwise stated. Statistical analyses were performed using SPSS statistics software version 21.0 (SPSS Inc. Chicago, United States). Comparison of the same parameter among groups was analyzed by one-way ANOVA. A value of *p* < 0.05 was considered to be statistically significant.

## Results

### Quantitative Analysis and Qualitative Composition of TAN

**FIGURE 1:**
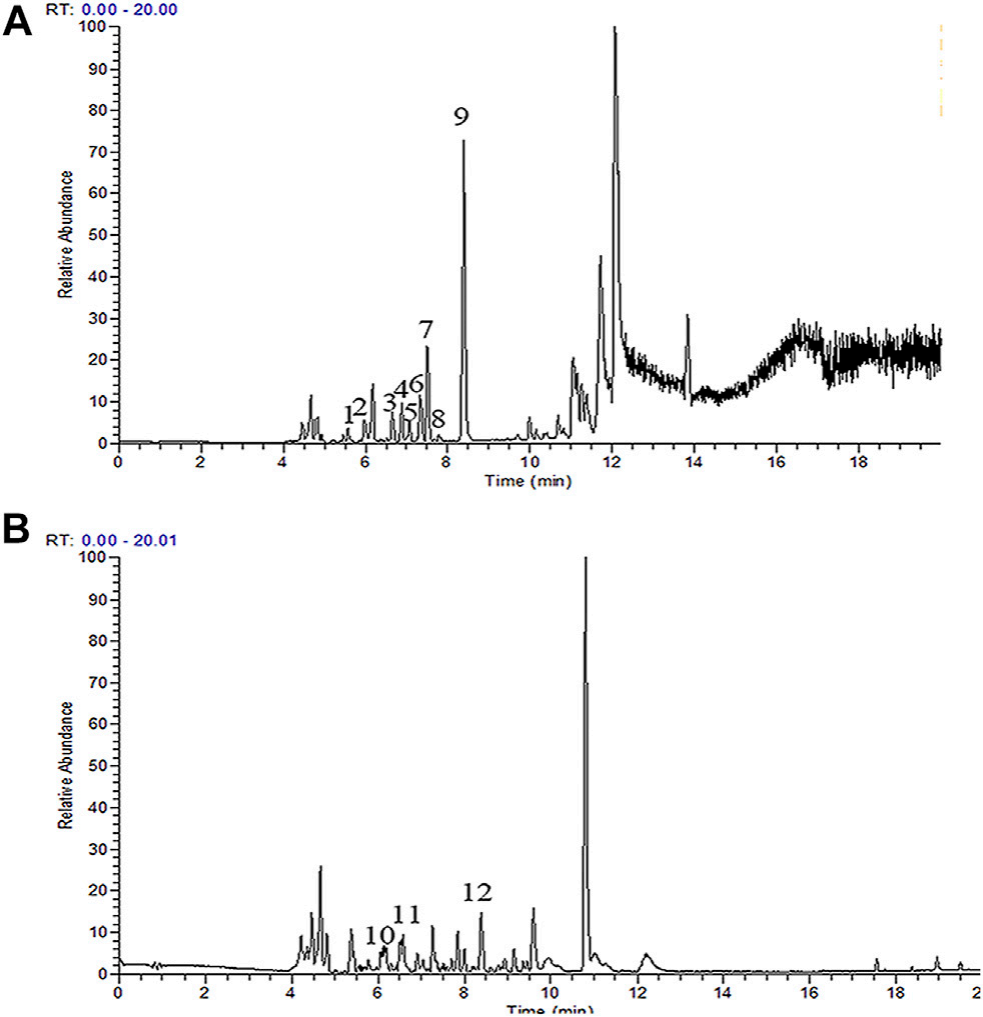
UHPLC-Orbitrap-MS spectrometry of TAN. Positive(A): Salvia miltiorrhiza new quinone B (1), Tanshinaldehyde(2), Dihydrotanshinone I(3), Tanshinone IIB (4), dehydromiltirone (5), Tanshinone Ⅰ(6), Cryptotanshinone (7), Methylenetanshinquinone (8), Tanshinone IIA (9), Negative(B):Danshin spiroketal lactone (10), Danshenxinkun A (11), Alpha-(3,4-dihydroxyphenyl)lactic acid (12).

UHPLC-Orbitrap-MS was used to characterize the chemical TAN composition. The total ion current chromatograms of the TAN are shown in Figure 1. Twelve major compounds were identified, including amounts of terpene like Salvia miltiorrhiza new quinone B, tanshinaldehyde, Dihydrotanshinone I, tanshinone IIB, dehydromiltirone, tanshinone I, cryptotanshinone, methylenetanshinquinone, tanshinone IIA, Danshin spiroketal lactone, Danshenxinkun A and a phenolic acid alpha-(3,4-dihydroxyphenyl)lactic acid. Details of the TAN compounds detected are shown in Supplementary Table S3.

**FIGURE 2:**
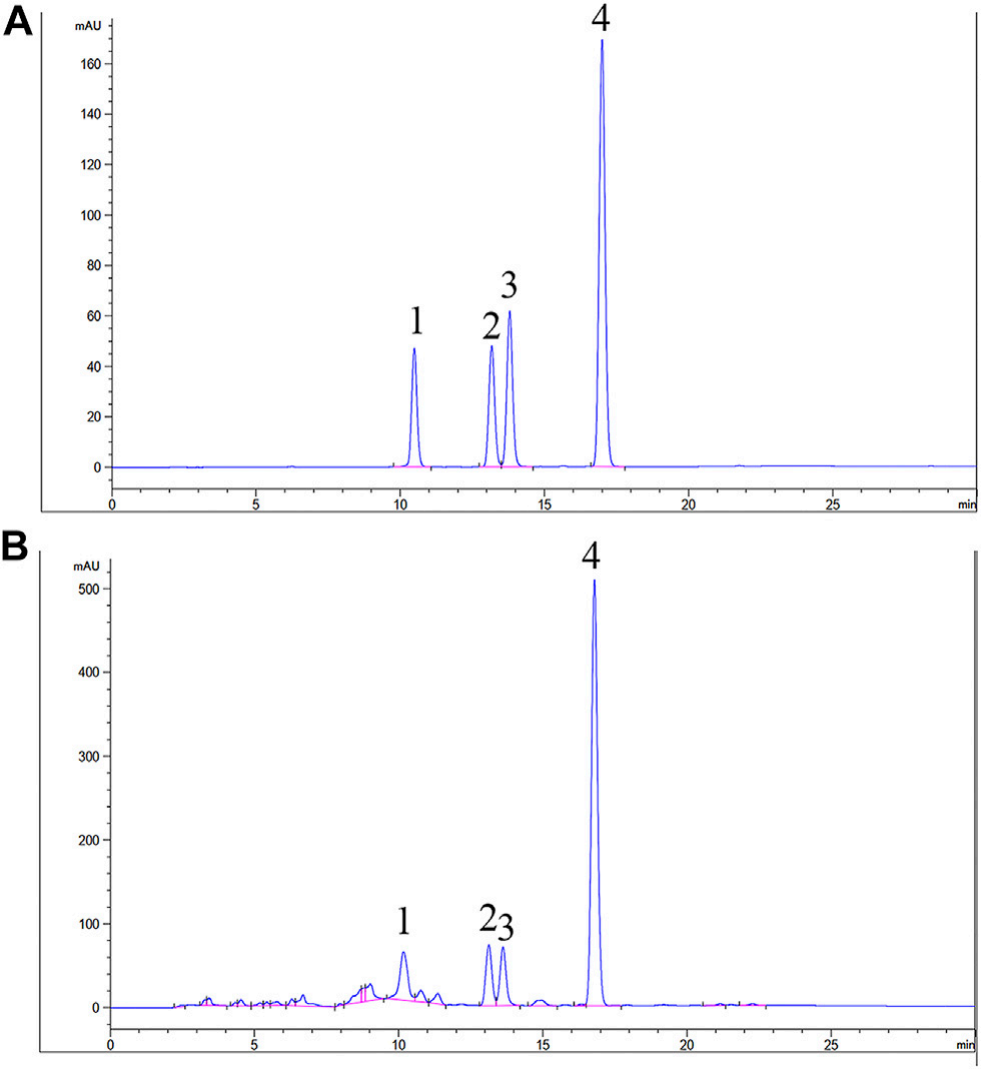
High-performance liquid chromatography chromatogram of A:standard mixture of 1:Diydrotanshinone Ⅰ, 2:Tanshinone Ⅰ, 3: Crytotanshinone, 4: Tanshinone IIA, B:TAN sample.

The TAN HPLC results are shown in Figure 2 and Table 1. All components were clearly distinguished at the retention time of 30 min. Dihydrotanshinone, Tanshinone I, crytotanshinone, and Tanshinone IIA were marked as markers of TAN, and were defined as 33.6, 27.8, 21.8, and 141.0 mg in 1 g TAN, respectively.

**TABLE 1:** Linear range, regression equation, coefficient of determination (r^2^) and amounts of marker compounds in TAN.

Compound	Linear range (µg/ml)	Regression equation	*r* ^2^	Amount (mg/g)	
				Mean	RSD (%)
Diydrotanshinone I	1.94–62.00	Y = 38.556X−2.2323	0.9999	33.6	1.08
Tanshinone I	3.13–100.00	Y = 38.377X−16.044	0.9996	27.8	0.06
Crytotanshinone	1.94–62.00	Y = 46.918X+18.035	0.9997	21.8	1.12
Tanshinone IIA	6.25–200.00	Y = 52.32X−16.816	0.9995	141.0	0.08

Y: peak area (mAU) of compounds; X: concentration (μg/ml) of compounds.

### Effect of TAN on Oleic Acid-Induced Acne in Rats

**FIGURE 3:**
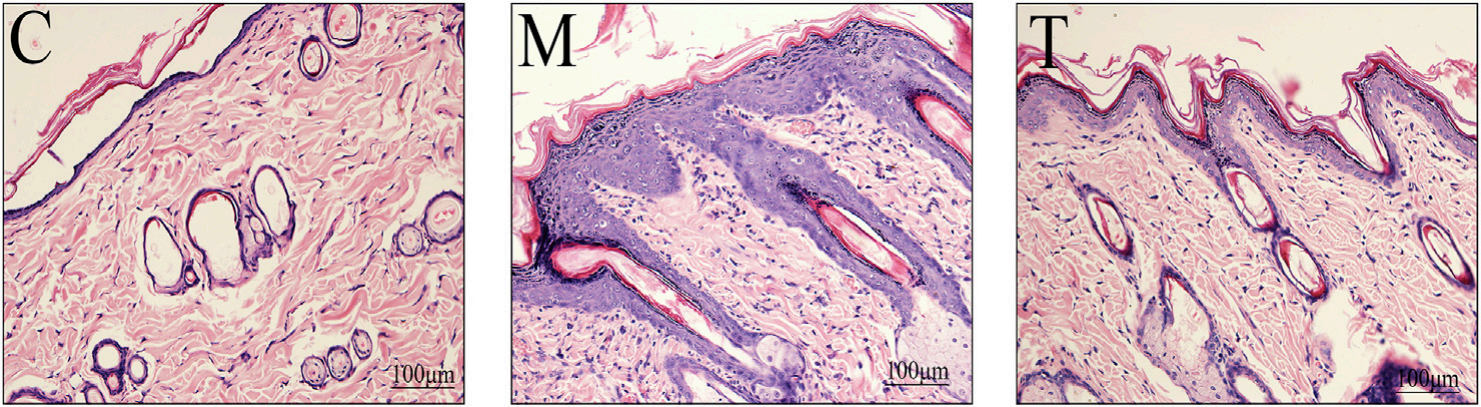
Skin histopathology among the three treatment groups (hematoxylin-eosin, 200 × magnification). C: control rats; M: oleic acid-induced acne rats; T: oleic acid-induced acne + TAN-treated rats.

The histological results are presented in Figure 3. In the M group, acne-like lesions developed on the back skin. Histological analysis of the skin revealed hyperplasia of the stratified squamous epithelium, which was accompanied by significant thickening of the stratum corneum. In the M group, dermis hyperemia, inflammatory cell infiltration, and sebaceous gland size were significantly increased (Figure 3A). Excessive keratinization of hair follicle sebaceous glands, blocking hair follicle pores, and a key pathological mechanism of acne development, was observed. M rats treated with TAN (Figure 3B) exhibited skin tissue similar to that of C rats, with improved keratosis, reduced inflammatory cell infiltration, and smaller sebaceous glands (Figure 3C).

**FIGURE 4:**
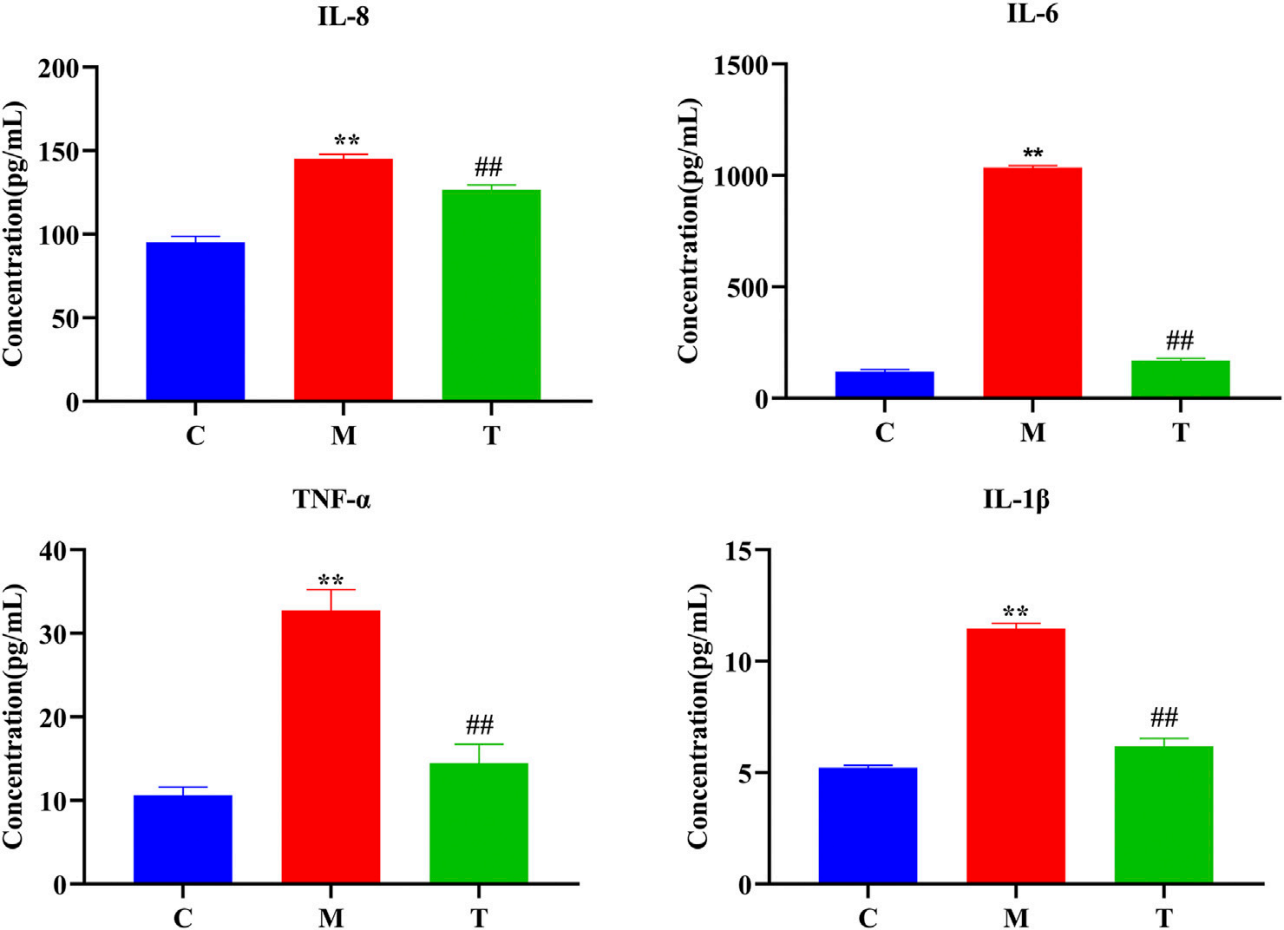
Expression levels of the serum inflammatory factors interleukin (IL)-8, IL-6, IL-β, and tumor necrosis factor alpha (TNF-α) (**, *p* < 0.01 vs. C rats; ##, *p* < 0.01 vs. M rats).

As illustrated in Figure 4, IL-8, IL-6, IL-β, and TNF-α expression were significantly higher in rats from the M group than the C and T groups (*p* < 0.05). The T group had similar serum cytokine levels to that of the C group, suggesting that TAN treatment was able to reverse high cytokine levels, present in the untreated M group, to levels exhibited by the C group (*p* < 0.05).

### Effect of TAN on Skin Lipidomics in Rats With Oleic Acid-Induced Acne

**FIGURE 5:**
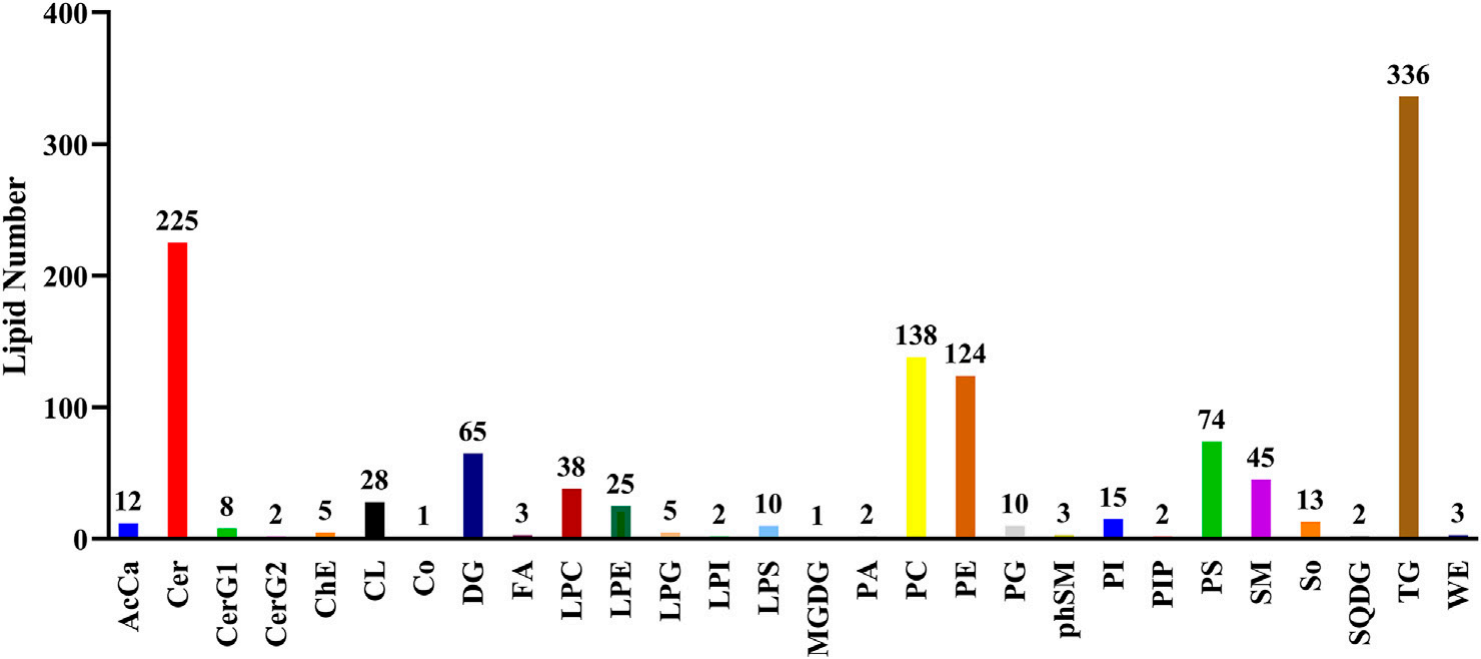
The number of lipid species within each identified lipid class based on. the International Lipid Classification and Nomenclature Committee.

According to the International Lipid Classification and Nomenclature Committee, lipid compounds can be divided into eight categories (Fahy et al., 2009). Each category can be sub-divided into different lipid classes based on polarity. Each class, based on differences in saturation or the length of the carbon chain, can be further subdivided into different molecular species (lipid species). Altogether, a three-level classification of lipid compounds is achieved. In this study, 28 lipid classes and 1,197 lipid species were identified. Figure 5 shows the number of lipid species in each lipid class.

**FIGURE 6:**
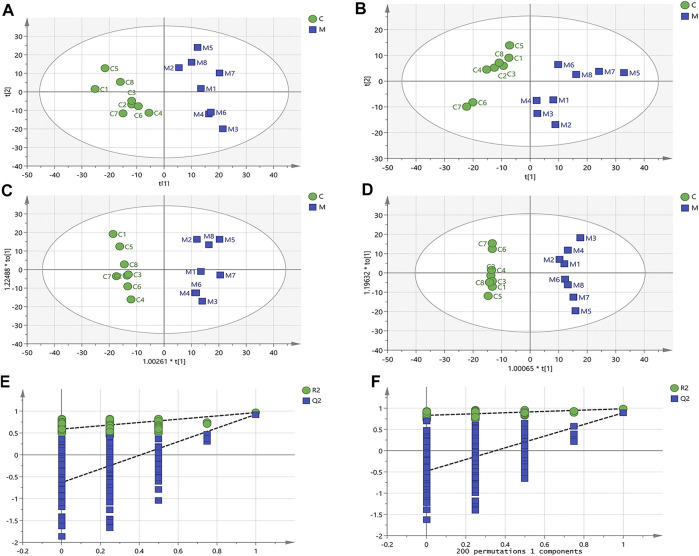
Multivariate data analysis of skin lipidomics. **(A)** The principal component analysis (PCA) score plots from the skin lipid profiles of the C and M experimental groups in positive ion mode (R^2^X = 0.746, Q2 = 0.531). **(B)** The PCA score plots from the skin lipid profiles of the C and M experimental groups in negative ion mode (R^2^X = 0.606, Q2 = 0.405). **(C)** The orthogonal partial least-squares discrimination analysis (OPLS-DA) score plots from the skin lipid profiles of the C and M experimental groups in positive ion mode (R^2^X = 0.609, R^2^Y = 0.964, Q2 = 0.924). **(D)** The OPLS-DA score plots from the skin lipid profiles of the C and M experimental groups in negative ion mode (R^2^X = 0.670, R^2^Y = 0.986, Q2 = 0.895). **(E)** Permutation test of the lipid species from the OPLS-DA model in positive ion mode (R^2^ = 0.576, Q2 = −0.623). **(F)** Permutation test of the lipid species from the OPLS-DA model in negative ion mode (R^2^ = 0.834, Q2 = −0.580).

According to the LipidSearch analysis, a visual data matrix was generated and exported to Simca 14.1 software for multivariate data analysis. PCA revealed the lipid changes in the C group compared to the M group. The results are illustrated in the score plots of Figures 6A,B. The model parameter (R^2^X), which indicates the explanatory power of a model, of Figures 6A,B, were 0.746 and 0.606, respectively.

OPLS-DA analysis demonstrated clear differentiation in lipidomic profiles between C and M groups as indicated in Figure 6C (R^2^X = 0.609, R^2^Y = 0.964, Q^2^ = 0.924) and Figure 6D (R^2^X = 0.670, R^2^Y = 0.986, Q^2^ = 0.895). Permutation testing and cross validation support this finding (Figure 6). Table 2 lists the significantly different lipids.

**TABLE 2:** 96 identified potential biomarkers among the C, M and T.

Lipid species	m/z	Rt (min)	C VS M		M^a^	T^b^
			*P*	Log2FC		
Negative						
Cer(d18:1/34:2)	830.7607	19.32406	0.0000	5.0295	↑	↓
Cer(d18:1/24:0)	726.6253	13.20895	0.0000	4.9656	↑	↓
Cer(d18:0/20:0)	670.5627	11.234	0.0000	4.5779	↑	↓
Cer(d42:2)	708.6148	13.82625	0.0000	3.9576	↑	↓
Cer(d18:0/20:0)	656.5835	12.71269	0.0003	3.7028	↑	↓
Cer(d18:0/21:0)	670.5991	12.89282	0.0000	3.6849	↑	↓
Cer(d17:0/25:0)	710.6304	15.03509	0.0000	3.5645	↑	↓
Cer(d54:1)	878.8182	20.47176	0.0014	3.4766	↑	↓
Cer(d18:0/26:0)	740.6774	15.76791	0.0007	3.4186	↑	↓
Cer(d18:0/23:0)	698.6304	13.9686	0.0000	3.3660	↑	↓
Cer(d18:1/30:1)	776.7137	17.99825	0.0000	3.2814	↑	↓
Cer(d52:1)	850.7869	19.29828	0.0000	3.2581	↑	↓
Cer(d18:1/28:1)	748.6824	16.714	0.0000	3.1965	↑	↓
Cer(d72:3)	1157.043	22.55396	0.0000	3.0347	↑	↓
Cer(d18:1/18:0)	642.5314	10.19465	0.0000	3.0183	↑	↓
Cer(d18:0/34:1)	850.7869	19.77001	0.0000	2.9937	↑	↓
Cer(d52:2)	848.7713	19.07259	0.0000	2.9919	↑	↓
Cer(d56:2)	904.8339	21.00001	0.0008	2.7741	↑	↓
Cer(d18:0/36:3)	874.7869	19.13745	0.0003	2.7521	↑	↓
Cer(d18:1/24:1?	708.6148	12.65092	0.0000	2.7243	↑	↓
Cer(d38:1)	654.5678	11.708	0.0000	2.6137	↑	↓
Cer(d50:1)	822.7556	18.55095	0.0000	2.5725	↑	↓
Cer(d18:1/32:1)	804.745	19.2463	0.0000	2.5524	↑	↓
Cer(d18:0/34:2)	848.7713	18.67903	0.0003	2.5181	↑	↓
Cer(d70:3)	1129.011	22.0871	0.0000	2.3984	↑	↓
Cer(d56:2)	904.8339	21.35417	0.0000	2.3947	↑	↓
Cer(d18:1/16:0)	614.5001	9.02884	0.0000	2.3678	↑	↓
Cer(d57:2)	918.8495	21.47109	0.0030	2.3654	↑	↓
Cer(d18:0/34:4)	844.74	16.90961	0.0008	2.3121	↑	↓
Cer(d16:1/26:1)	708.6148	12.9112	0.0000	2.2731	↑	↓
Cer(d18:1/18:0)	626.5365	10.70592	0.0000	2.1982	↑	↓
Cer(d59:3)	944.8652	21.84285	0.0035	2.0980	↑	↓
Cer(d55:2)	890.8182	20.85184	0.0000	2.0728	↑	↓
Cer(d60:4)	956.8652	21.56007	0.0001	2.0672	↑	↓
Cer(d18:0)	820.74	17.66335	0.0003	2.0053	↑	↓
FA (20:5)	301.2173	2.033	0.0049	2.5654	↑	↓
LPE (20:0)	508.3409	5.446,271	0.0000	2.0328	↑	↓
PE (16:1/18:2)	712.4923	7.434,218	0.0001	2.9635	↑	↓
PE (18:0/18:2)	742.5392	9.448,973	0.0004	2.5266	↑	↓
PE (24:2/18:2)	822.6018	12.57836	0.0000	2.0977	↑	↓
PE (36:5)	736.4923	7.194,899	0.0100	2.0491	↑	↓
Positive						
DG (15:0)	348.2745	1.3521	0.0000	6.6497	↑	↓
AcCa(20:4)	448.3421	2.1250	0.0000	2.3769	↑	↓
AcCa(18:2)	424.3421	2.1920	0.0001	2.2946	↑	↓
Cer(d18:1/24:0)	666.6395	14.8970	0.0000	3.7160	↑	↓
Cer(d36:1)	566.5507	13.2694	0.0000	3.5810	↑	↓
Cer(d40:1)	622.6133	15.5625	0.0001	3.3591	↑	↓
Cer(d18:0/24:0)	668.6551	14.7050	0.0016	3.2196	↑	↓
Cer(d18:0/16:0)	572.5249	10.1544	0.0000	3.0980	↑	↓
Cer(d18:1/54:0)	1101.088	24.5525	0.0000	3.0138	↑	↓
Cer(d18:1/16:1)	552.4986	8.8579	0.0000	2.6150	↑	↓
Cer(d50:1)	794.7596	13.5360	0.0000	2.5911	↑	↓
Cer(d18:1/24:0)	666.6395	13.7101	0.0000	2.5894	↑	↓
Cer(d34:1)	538.5194	12.2471	0.0002	2.5210	↑	↓
Cer(d50:2)	776.749	14.0652	0.0000	2.4725	↑	↓
Cer(d18:1/56:0)	1129.12	24.8739	0.0000	2.4222	↑	↓
Cer(d18:0/22:0)	640.6238	13.6112	0.0000	2.3973	↑	↓
Cer(d18:0/17:0)	586.5405	10.6643	0.0015	2.2800	↑	↓
Cer(d18:2/51:2)	1052.994	22.1339	0.0000	2.2269	↑	↓
Cer(d44:1)	678.6759	17.9162	0.0001	2.0399	↑	↓
Cer(d18:1/26:0)	694.6708	14.8389	0.0000	2.0371	↑	↓
DG (36:2p)	622.5769	13.3210	0.0001	2.5883	↑	↓
DG (22:0/18:2)	694.6344	15.5480	0.0000	2.5602	↑	↓
DG (24:0/18:2)	722.6657	16.7877	0.0000	2.4717	↑	↓
DG (24:1/18:2)	720.6501	15.4434	0.0000	2.0170	↑	↓
LPC(24:0)	608.465	9.0185	0.0000	2.2503	↑	↓
So(d18:0)	302.3054	2.6342	0.0000	3.4427	↑	↓
TG (30:1/20:0/22:4)	1151.068	25.0762	0.0001	3.2637	↑	↓
TG (26:1/24:1/24:2)	1181.114	25.6011	0.0001	2.8942	↑	↓
TG (30:1/18:1/22:3)	1123.036	24.7924	0.0000	2.8787	↑	↓
TG (67:4)	1083.005	21.1760	0.0000	2.8079	↑	↓
TG (30:1/22:2/24:1)	1209.146	25.8468	0.0001	2.7197	↑	↓
TG (30:1/18:2/24:1)	1153.083	25.3393	0.0002	2.6218	↑	↓
TG (30:1/24:1/24:2)	1237.177	26.0781	0.0002	2.2441	↑	↓
TG (24:0/20:4/24:1)	1095.005	24.4620	0.0000	2.1535	↑	↓
TG (30:1/18:2/22:1)	1125.052	25.0774	0.0003	2.0958	↑	↓
TG (16:0/12:1/18:2)	790.6919	16.9359	0.0001	−2.0249	↑	↓
TG (15:0/16:0/16:1)	808.7389	19.7909	0.0002	−2.0465	↑	↓
TG (16:0/14:0/18:3)	818.7232	18.1460	0.0001	−2.0516	↑	↓
TG (8:0/8:0/24:1)	710.6293	14.7050	0.0066	−2.0949	↑	↓
TG (4:0/16:0/18:0)	684.6137	15.8794	0.0019	−2.0951	↑	↓
TG (15:0/16:0/16:0)	810.7545	20.7548	0.0001	−2.1094	↑	↓
TG (6:0/16:0/16:0)	684.6137	15.5811	0.0033	−2.1311	↑	↓
TG (16:0/8:0/18:1)	738.6606	16.7619	0.0003	−2.1414	↑	↓
TG (16:0/16:1/18:2)	846.7545	19.3644	0.0001	−2.1634	↓	↑
TG (16:0/14:0/16:0)	796.7389	20.3731	0.0001	−2.1929	↓	↑
TG (16:0/12:1/16:0)	766.6919	18.3072	0.0003	−2.1941	↓	↑
TG (8:0/18:1/18:2)	762.6606	15.7967	0.0019	−2.2486	↓	↑
TG (6:0/18:1/18:2)	734.6293	14.7557	0.0006	−2.2570	↓	↑
TG (16:0/12:1/16:0)	766.6919	17.9574	0.0002	−2.2812	↓	↑
TG (16:0/14:1/16:0)	794.7232	19.1828	0.0002	−2.3662	↓	↑
TG (4:0/16:0/16:0)	656.5824	14.6726	0.0038	−2.4827	↓	↑
TG (16:0/14:0/18:1)	822.7545	20.3946	0.0001	−2.4857	↓	↑
TG (6:0/16:0/16:1)	682.598	14.6957	0.0031	−2.6041	↓	↑
TG (6:0/16:0/18:1)	710.6293	15.6969	0.0024	−2.8806	↓	↑
TG (4:0/16:0/16:1)	654.5667	13.5966	0.0007	−3.6163	↓	↑

(↑):Upregulated (*p* < 0.05, *n* = 8) (↓): Downregulated (*p* < 0.05, *n* = 8).

a Trends of the M group compared with the C group of the metabolites.

b Trends of the T group compared with the M group of the metabolites.

A total of 96 lipid species, 55 and 41 in positive and negative ion mode, respectively, were identified. In negative ion mode, the concentrations of 35 ceramides (Cers), 4 phosphatidylethanolamines (PEs), 1 lysophosphatidyl ethanolamine (LPE), and 1 fatty acid (FA) were significantly increased (*p* < 0.05) in the M group compared to the C group. In positive ion mode, the concentrations of 17 Cers, 2 acyl carnitines (AcCas), 5 diglycerides (DGs), 1 lysophosphatidylcholine (LPC), 1 So, and 9 triglycerides (TGs) were significantly increased, while the concentrations of 12 TGs were significantly decreased (*p* < 0.05), in the M group compared to the C group.

**FIGURE 7:**
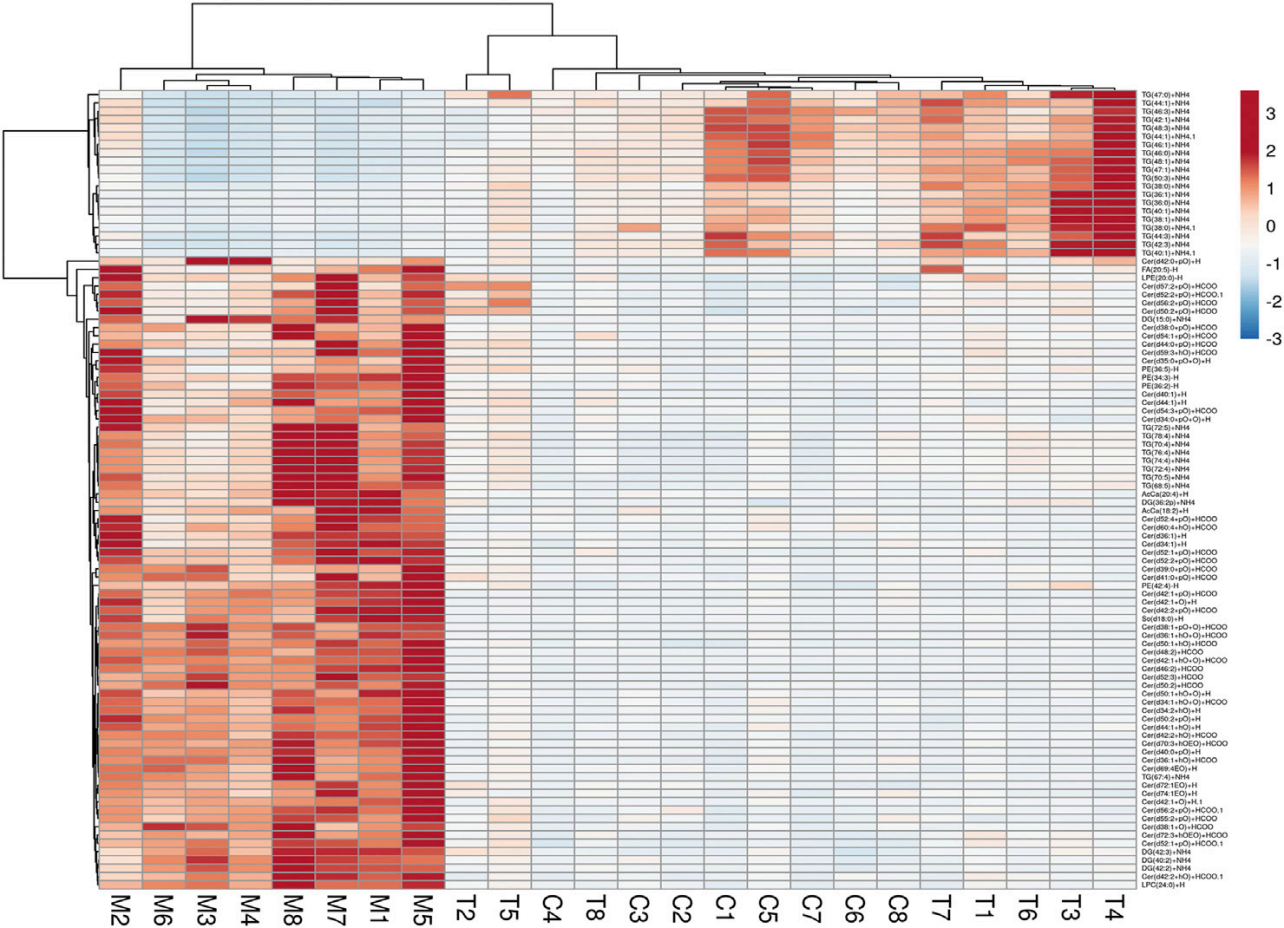
Heatmap of 96 lipid species among the experimental groups. Each line of this graph represents an accurate mass ordered by the retention time and is colored by its abundance intensity. The scale from −3 blue (low) to + 3 red (high) represents the abundance.

To further elucidate the metabolic differences between the C and M groups, the identified lipids were analyzed using a clustering heatmap. Significantly different lipids between M and C groups were considered to be potential biomarkers (Figure 7). The heatmap in Figure 7 directly expresses the variability of each lipid species, and illustrates their relative increase (red) or decrease (blue) in M rats compared to the C and T groups, which leads us to postulate that TAN can improve the disease state as shown in Figure 3.

**FIGURE 8:**
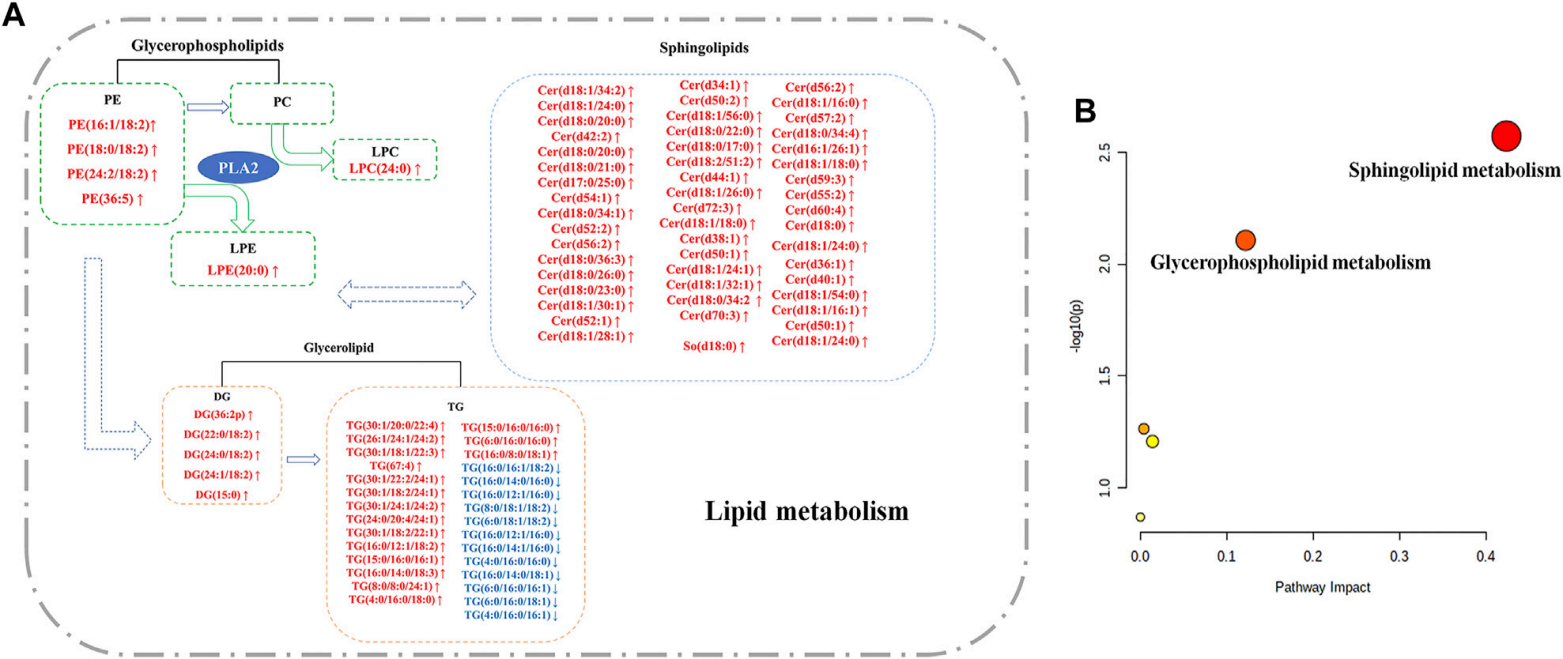
Lipid metabolic pathway analysis of identified differential lipid species. **(A)** The network of potential biomarker variation in M rats compared to C rats in Lipid Metabolism. Red (↑): upregulation; Blue (↓): downregulation. **(B)** The Metabolomics Pathway Analysis identified sphingolipid metabolism, glycerophospholipid metabolism, and linoleic acid metabolism from the significantly differential lipid species. The size and color of each circle are based on pathway impact values and *p*-values, respectively.

The lipid metabolism pathway analysis was performed using Metabolomics Pathway Analysis (MetPA) 5.0. A total of 96 identified metabolites were mapped to the Human Metabolome Database to obtain IDs to perform pathway enrichment analysis. The differential lipid species were analyzed using MetPA, and the results are shown in Table 3. Impact values >0.01 and *p*-values < 0.05 were considered as the screening conditions. Figure 8 demonstrates that sphingolipid and glycerophospholipid metabolism had the highest impact factors.

**TABLE 3:** Ingenuity pathway analysis with MetPA from differential lipid species.

Pathway name	Match status	*P*	−log(p)	Holm p	FDR	Impact	KEGG
Sphingolipid metabolism	2/21	0.0026767	2.5724	0.22484	0.22484	0.42394	00600
Glycerophospholipid metabolism	2/36	0.0078181	2.1069	0.6489	0.32836	0.12185	00564
Glycosylphosphatidylinositol (GPI)-anchor biosynthesis	1/14	0.054479	1.2638	1.0	1.0	0.00399	00563
Glycerolipid metabolism	1/16	0.062056	1.2072	1.0	1.0	0.01402	00561

## Discussion

The therapeutic effects of TAN on acne were observed in a rat model of oleic acid-induced acne. Our results indicated an upregulation of 84 lipid species and a downregulation 12 lipid species in M rats compared to C rats. This is in agreement with previous metabolome database reports of a dysregulation in lipid species, in particular sphingolipid and glycerophospholipid metabolism, as a sign of acne (Camera et al., 2016).

### Change of Sphingolipid Metabolism

In this study, we found that Cer concentrations in the M group were increased, suggesting that Cers were more active in the M rats than those in the C group. Among them, concentrations of Cer(d42:1), Cer(34:0), and Cer(34:1), ultra-long chain Cers, were significantly increased. The altered Cer expression profiles may lead to decreased extracellular lipid matrix density and increased risk of exogenous invasion, which in turn stimulates the Th2/Th22 inflammatory response (Li et al., 2020). In addition, disturbed Cer expression profiles, and impaired barrier function, result in ongoing production of cytokines and chemokines, such as IL-1α, TNF-α, and β-defensins, that exacerbate the inflammatory response (Kanoh et al., 2019). In skin, Cers regulate the balance between keratinocyte proliferation and differentiation by exerting anti-proliferative and pro-apoptotic effects. Increased Cer synthesis, along with increased rates of keratinocyte differentiation, have been detected *in vitro* and *in vivo* models (Mizutani et al., 2013). We postulate that these increases may lead to corneum thickening in the M group, compared to the C and T groups. The role of Cers and its derivatives in regulating immune responses has been extensively studied. Cers are considered bioactive transmitters that are involved in various inflammatory signaling pathways. Further research is needed to determine whether extracellular Cer accumulating in the lipid matrix of the stratum corneum is also involved in inflammation and the immune response in acne. Notably, Cers can be broken down by ceramidase to produce sphingosine and FAs.

### Change of Glyceride Metabolism

DGs are secreted by sebaceous glands and help maintain skin barrier stability. DGs are also second messengers involved in the inflammatory response (Grkovich and Dennis, 2009). TGs are formed by the combination of DGs and FAs by glyceryl diesteryl transferase. TGs on the skin surface can be degraded by resident skin bacteria to generate DGs and free FAs (Camera et al., 2016). DGs result from the hydrolysis of TGs. In this study, variations in DG and TG differential metabolites in M compared to C and T groups indicates their dysregulation, suggesting another area of investigation for acne treatment.

The synthesis of TGs *in vivo* involves two main pathways: mono-glycerol and diglycerol synthesis. TGs are hydrolyzed into glycerol and FAs by a series of lipases, which then undergo β-oxidation for absorption and use by the body. TG hydrolysis requires catalysis by hormone-sensitive lipases which exist in two main forms: short- and long-chain forms. The short-chain form is mainly expressed in adipose tissue and catalyzes the hydrolysis of TGs into FAs. The long-chain form is expressed in steroidal tissues, such as the testes, and hydrolyzes cholesterol esters into free cholesterol, which is then converted, by a series of enzymatic reactions, into androgens and estrogens.

In this study, 29 different TG compounds were identified, with 58.6% were found in significantly higher concentrations in C rats compared to M rats. However, according to the TG-omics analysis, TGs had fewer differential metabolites, with an inconsistent variation trend, in contrast to the DG results. The enzymes catalyzing TG hydrolysis may therefore be dysfunctional in the M rats. For glycerolipids, TG accumulation in non-adipose tissue is associated with lipid toxicity (Liu et al., 2012). In the M group, abnormal fat infiltration was observed, which could have contributed to increased lipid toxicity and acne initiation in those rats.

### Change of Fatty Acid Metabolism

FAs are the most basic unit and metabolite of lipid metabolism; its structure and intracellular concentrations have important implications for lipid metabolism in cells. In our study, it was found that DGs and FAs exhibited a uniform increasing or decreasing trend in model group rats.

In biological systems, carnitine can combine with FAs to form AcCa, which promotes the transport of FAs to the mitochondrial intima for fatty acid β-oxidation.

### Change of Glycerylphospholipid Metabolism

Glycerylphospholipids, such as phosphatidylcholine (PC), PE, LPC, lysophosphatidic acid (LPA), and LPE, are components of all cell membranes. Typical phospholipid molecules consist of hydrophilic phosphate heads and two lipophilic (hydrophobic) fatty chains. These lipids induce intracellular signal transduction by activating G-protein-coupled receptors on the cell membrane. Consequently, they play important biological functions in embryogenesis, cell proliferation, lymphocyte homing, and the inflammation-induced immune response. The lipid biomarkers obtained in this study were mainly from the LPC, PE, and LPE subgroups.

LPC is produced by phospholipase A2-mediated hydrolysis of PC, as depicted in Figure 8. LPC is a biologically active lipid that can be produced under pathological conditions. It is an amphoteric molecule that functions as both a surfactant and a detergent. LPC content is regulated to maintain proper cellular activity, as it easily damages cells at high concentrations; for example, through weakening the integrity of monocytes and smooth muscle cells. As a member of the stain remover family, LPC can cause cell lysis at higher concentrations, while at lower concentrations can change membrane permeability. LPC has been found to injure endothelial cells in the human umbilical vein. Moreover, it has also been reported to be involved in various pathological conditions such as diabetes, obesity, atherosclerosis, and cancer (Kabarowski et al., 2005). We therefore postulate that this would be a new biomarker to promote the occurrence of acne.

In the heathly human body, activated ethanolamine combines with DGs to produce PE. LPE is a metabolite generated by the enzymatic hydrolysis of PE by phospholipase A1. The PE-LPE metabolic pathway is involved in various cellular metabolic pathways (Shirvan et al., 2017). These aforementioned phospholipids play a role in several metabolic pathways in the body, including those involving the metabolism of glycerolipids, arachidonic acids, linoleic acids, α-linoleic acids; integrative metabolism; and the retrograde endocannabinoid signaling pathway. During phospholipid synthesis and metabolism, lipid metabolites are produced *via* the action of enzymes. Under normal physiological conditions, the amount of these lipid metabolites is regulated, but under an inflammatory state, they can aggregate and produce pathological effects. Many lipid metabolites are also biologically active secondary messengers and, in many cases, are associated with the onset of disease. The pathways involved in glycerolipid metabolism imply that LPCs, PEs, and LPEs can be converted into one another, as illustrated in Figure 8. In conclusion, we speculate that the uncontrolled lipid species can be a warning signal of acne.

## Conclusion

To summarize, using lipidomic analysis, this study identified 96 different lipid species from the sphingolipid and glycerophospholipid metabolism pathways in an oleic acid-induced acne model. Our results suggest that TAN may effectively treat acne by regulating the metabolism of lipids, such as phospholipids and sphingolipids. In addition, lipidomics may be useful in investigating the effects, and explaining possible mechanisms of action, of other traditional medicinal plants on skin diseases.

## Data Availability

The raw data supporting the conclusion of this article will be made available by the authors, without undue reservation, to any qualified researcher. Requests to access these datasets should be directed to liuqiang@smu.edu.cn.

## Glossary

AcCa: acyl carnitine
Cer: Ceramide
chE: cholestery1 ester
CL: cardiolipin
Co: coenzyme
DG: diglyceride
FA: fatty acid
HPLC: High-performance liquid chromatography chromatogram
IL-1β: interleukin-1β
IL-6: interleukin-6
IL-8: interleukin-8
LC-MS: liquid chromatography-mass spectrometry
LPC: lysophosphatidylcholine
LPE: lysophosphatidyl ethanolamine
LPI: lysophosphatidylinositol
LPG: lysophosphatidylglycerol
LPS: lysophosphatidylserine
MGDG: monogalactosyldiacylglycerol
PA: phosphatidic acid
PC: phosphatidylcholine
PCA: principal component analysis
PE: phosphatidylethanolamine
PG: phosphatidylglycerol
phSM: phytosphingosine
PI: phosphatidylinositol
PS: phosphatidy1serines
QC: quality control
SM: sphingomyelin
So: sphingosine
SQDG: sulfoquinovosyldiacylglycerol
TG: triglyceride
TNF-α: tumor necrosis factor α
VIP: variable importance on projection
WE: wax exters
