# Supplementary material for: A Skin Lipidomics Study Reveals the Therapeutic Effects of Tanshinones in a Rat Model of Acne
Source: Front Pharmacol. 2021 Jun 10;12:675659. doi: 10.3389/fphar.2021.675659 (PMC8223585; doi:10.3389/fphar.2021.675659)
Supplement: Supplementary file 2 [file DataSheet1.docx]

Supplementary Material

# Supplementary Data

# Methods

## 1.1 The TAN standard solutions preparation

The reference substances of Dihydrotanshinone Ⅰ, cryptotanshinone, tanshinone І, tanshinone ⅡA were precisely weighed and added with methanol to yield concentration of 1.9375-62 μg·mL^-1^, 1.9375-62 μg·mL^-1^, 3.125-100 μg·mL^-1^, 6.25~200 μg·mL^-1^ respectively.

## 1.2 The analysis of TAN by UHPLC-ESI-MS

Separation was performed using an Vanquish UHPLC (Thermo Scientific, USA) equipped with a UHPLC Hypersil Gold analytical column (2.1 mm×100 mm, 1.9 μm)(Thermo Scientific, USA). The extractive were eluted using buﬀer A (0.01 % formic acid-water) and buffer B (0.01% formic acid- methanol), at a flow rate of 300 μL/min. The gradient are as followed: 0-4 min buffer A from 80 % to 25 %, 4-9min buffer A from 25 % to 15 %; 9-13 min buffer A hold 15 %, 13-16 min from 15 % to 0 %; then in16-20 min buffer A hold 0 %.

The MS was performed on a Orbitrap Fusion system (Thermo Scientific, USA) operating in an ESI in both positive and negative modes (positive ion 3500 V negative ion 3000 V). Orbitrap resolution was 60,000 with scan range of 100-1000 (*m/z*). The nebulization gas was set to 40 Arb at a temperature of 350 ℃, and the Curtain gas was set to 10 Arb. For the MS2, cycle time data dependent mode (DDA) was used to acquire data. The fragmentation ions were further fragmented using HCD, the resulting reporter ions were used for quantification, the HCD collision energy is 30 %, and detection type was iron trap with isolation window 1.6 (m/z).

## 1.3 The analysis of TAN by HPLC

HPLC (Agilent Technologies, Santa Clara, CA, United States) with an UV detection system was used for quantitative analysis. TANs were separated by a Nanologica SVEA C18 column (250×4.6 mm, 5 μm). The injection volume was 10μL. The mobile phase consisted of linear gradients of acetonitrile (A) and 0.026 % (*v/v*) phosphoric acid (B) : 0–20 min, 60-90 % A (v/v), 40-10 % B (v/v); 20-30 min, 90 % A, 10% B; The mobile phase flow rate was 1.0 mL·min^-1^. The column was run at 25 °C. The detection wavelength was 270 nm.

## 1.4 The skin lipid species by UHPLC-ESI-MS/MS

Reverse phase chromatography was selected for LC separation using CSH C18 column (1.7 um, 2.1 mm×100 mm, Waters). The lipid extracts were re-dissolved in 200 uL 90 % isopropanol/acetonitrile, centrifuged at 14000 g for 15 min, and 3 uL of sample was injected. Solvent A was acetonitrile-water (6:4, v/v) with 0.1% formic acid and 0.1 mM ammonium formate and solvent B was acetonitrile-isopropanol (1:9, v/v) with 0.1 % formic acid and 0.1 mM ammonium formate. The initial mobile phase was 30 % solvent B at a flow rate of 300 uL·min^-1^. It was held for 2 min, and then linearly increased to 100 % solvent B in 23 min, followed by equilibrating at 5 % solvent B for 10 min. Mass spectra was acquired by Q-Exactive Plus in positive and negative mode, respectively. ESI parameters were optimized and preset for all measurements as follows: Source temperature, 300 °C; Capillary Temp, 350 °C, the ion spray voltage was set at 3000 V, S-Lens RF Level was set at 50 % and the scan range of the instruments was set at m/z 200-1800.

# Supplementary Tables

## 2.1 Supplementary Table S1. Mobile phase composition for UHPLC.

| **Time** (min) | **A** (%) | **B** (%) |
| --- | --- | --- |
| 0-4 | 80-25 | 20-75 |
| 4-9 | 25-15 | 75-85 |
| 9-13 | 15 | 85 |
| 13-16 | 15-0 | 85-100 |
| 16-20 | 0 | 100 |

## 2.2 Supplementary Table S2. Mobile phase composition for HPLC.

| **Time** (min) | **A** (%) | **B** (%) |
| --- | --- | --- |
| 0~20 | 60~90 | 40~10 |
| 20~30 | 90 | 10 |

## 2.3 Supplementary Table S3. TAN compounds identified by UHPLC-Orbitrap-MS.

| **NO.** | **t_R_/min** | **Adduct** | **Formula** | **MS/MS fragments** | **Compound** |
| --- | --- | --- | --- | --- | --- |
| 1 | 5.74 | [M+H] | C18H16O3 | 281.03[M+H]、253.09[M+H-CO]、235.08[M+H-CO-H2O] | Salvia miltiorrhiza new quinone B |
| 2 | 6.04 | [M+H] | C19H17O4 | 310.12[M+H]、282.12[M+H-CO] | Tanshinaldehyde |
| 3 | 6.7 | [M+H] | C18H14O3 | 279.10[M+H]、261.09[M+H-H2O]、233.10[M+H -H2O-CO] | DihydrotanshinoneⅠ |
| 4 | 6.92 | [M+H] | C19H18O4 | 311.13[M+H]、293[M+H-H2O]、275[M+H-H2O-H2O]、278[M+H-H2O-CH3]、283[M+H-CO]、247[M+H-H20-CO-H2O] | TanshinoneⅡB |
| 5 | 7.02 | [M+H] | C19H20O2 | 281.12[M+H]、263.11[M+H-H2O]、235.11[M+H-H2O-CO] | Dehydromiltirone |
| 6 | 7.38 | [M+H] | C18H12O3 | 277.09[M+H]、249.09[M+H-CO] | TanshinoneⅠ |
| 7 | 7.55 | [M+H] | C19H20O3 | 297.15[M+H]、279.14[M+H-H2O]、251.14[M+H-H2O-CO] | Cryptotanshinone |
| 8 | 7.82 | [M+H] | C18H14O3 | 279.10[M+H]、261.09[M+H-H2O]、233.10[M+H-H2O-CO]、205.10[M+H-H2O-CO-H2O]、190.08[M+H-H2O-CO-H2O-CH3] | Methylenetanshinquinone |
| 9 | 8.42 | [M+H] | C19H18O3 | 295.13[M+H]、277.12[M+H-H2O]、266.09[M+H-C2H5]、253.09[M+H-C3H6]、249.13[M+H-H2O-CO] | TanshinoneⅡA |
| 10 | 6.35 | [M-H] | C18H22O3 | 285.15[M-H]、257.15[M-H-CO]、211.15[M-H-CO-CO-CO] | Danshin spiroketal lactone |
| 11 | 6.62 | [M-H] | C18H16O4 | 295.10[M-H]、277.09[M-H-H2O]、265.09[M-H-C2H6] | Danshexinkun A |
| 12 | 8.48 | [M-H] | C18H16O4 | 295.13[M-H]、280.11[M-H-CH3] | Alpha-(3,4-dihydroxyphenyl)lactic acid |

## 2.4 Supplementary Table S4. Standard curve, R2, linear range of components in TAN by HPLC.

| **Analyst** | **Content**  (μg·mL^-1^) | **Peak Area**  **(**mAU) | **Standard curve** | **R^2^** |
| --- | --- | --- | --- | --- |
| Dihydrotanshinone Ⅰ | 1.9375 | 76.5 | y=38.556x-2.2323 | 0.9999 |
|  | 3.875 | 152.2 |  |  |
|  | 7.75 | 286.7 |  |  |
|  | 15.5 | 605.4 |  |  |
|  | 31 | 1178 |  |  |
|  | 62 | 2394 |  |  |
| Cryptotanshinone | 1.9375 | 95.6 | y=46.918x+18.035 | 0.9997 |
|  | 3.875 | 197.4 |  |  |
|  | 7.75 | 397.1 |  |  |
|  | 15.5 | 728.7 |  |  |
|  | 31 | 1500.7 |  |  |
|  | 62 | 2915.6 |  |  |
| Tanshinone І | 3.125 | 116.5 | y=38.377x-16.044 | 0.9996 |
|  | 6.25 | 244.3 |  |  |
|  | 12.5 | 479.4 |  |  |
|  | 25 | 898.9 |  |  |
|  | 50 | 1879.2 |  |  |
|  | 100 | 3841 |  |  |
| Tanshinone ⅡA | 6.25 | 369.5 | y=52.32x-16.816 | 0.9995 |
|  | 12.5 | 694.6 |  |  |
|  | 25 | 1324 |  |  |
|  | 50 | 2450.7 |  |  |
|  | 100 | 5157.9 |  |  |
|  | 200 | 10503.4 |  |  |
